# Examining psychosocial correlates of physical activity and sedentary behavior in youth with and without HIV

**DOI:** 10.1371/journal.pone.0225890

**Published:** 2019-12-02

**Authors:** Leapetswe Malete, Dawn M. Tladi, Jennifer L. Etnier, Jerry Makhanda, Gabriel M. Anabwani

**Affiliations:** 1 Michigan State University, East Lansing, United States of America; 2 University of Botswana, Gaborone, Botswana; 3 University of North Carolina Greensboro, Greensboro, United States of America; 4 The Botswana-Baylor Children’s Clinical Centre of Excellence, Gaborone, Botswana; The Education University of Hong Kong, HONG KONG

## Abstract

The objectives of this study were to examine differences in physical activity behaviors as a function of human immunodeficiency virus (HIV) status and sex, to test differences in physical activity self-efficacy (PASE), body weight satisfaction (BWS), and enjoyment of physical activity as a function of HIV status, and to determine if PASE, BWS, and enjoyment are associated with daily physical activity (daily PA), muscle strengthening activities, and sedentary behavior of youth with and without HIV. A total of 250 HIV positive (HIV+) and HIV negative (HIV-) youth from Botswana aged 12–23 years (Mean = 17.87, SD = 2.24) participated in the study. The HIV+ group (n = 88) was recruited from a previous 12-month antiretroviral therapy (ART) and nutrition intervention study. The HIV- group (n = 162) was randomly selected from public junior and senior (secondary) high schools in and around Gaborone. Participants’ PASE, BWS, enjoyment of physical activity, daily PA, muscle strengthening, body mass index (BMI), and sedentary behavior were obtained using items from the Youth Risk Behavior Surveillance Survey. Multivariate analysis of variance (MANOVA) showed that the HIV- group (*M* = 1.20, *SE* = 0.06, *CI* = 1.08 to 1.32) had significantly higher daily PA than the HIV+ group (*M* = 0.99, *SE* = 0.08, *CI* = 0.82 to 1.15). The HIV- group (*M* = 0.91, *SE* = 0.06, *CI* = 0.79 to 1.03) also reported participating significantly more in muscle strengthening activities than the HIV+ group (*M* = 0.63, *SD* = 0.08, *CI* = 0.47 to 0.78). Multiple regression analyses showed that higher PASE (*p <* .001) and greater enjoyment of PA (*p <* .01) were predictive of higher daily PA. HIV- participants had higher PASE but lower BWS compared to HIV+ participants. Sex and age differences were observed in muscle strengthening activities and sedentary behavior. This study supports previous findings on the association of efficacy beliefs to daily PA and muscle strengthening activities. The findings have implications for PA interventions aimed at health promotion and mitigation of the effects of living with HIV/AIDS.

## Introduction

Self-efficacy is one of the most studied psychological correlates of physical activity (PA) and exercise behaviors [1–3]. A key construct of Bandura’s social learning theory [4,5], self-efficacy is defined as the confidence individuals possess about their capacity to execute a course of action even under challenging circumstances. Efficacy beliefs are influenced by both controllable and uncontrollable factors such as task mastery, role modeling, social support, and reinforcement of behaviors as well as self-evaluations of skills and competencies [4]. Using this framework, studies have reported a reciprocal relationship between children and adolescents’ confidence in their motor abilities and capacity to engage in physical activities [6,7]. Specifically, physical activity self-efficacy (PASE) has been associated with increased and long-term participation in PA [8–11]. Other variables that have been associated with PA participation in children and youth are enjoyment of physical activity (enjoyment of PA) and body weight satisfaction (BWS) [8,12,13,14].

Enjoyment of PA relates to intrinsic motives, such as moods, feelings, and attitudes, including the sheer pleasure of participation in PA [14]. Enjoyment of PA has been reported in the literature as one of the leading determinants of PA in children and adolescents [15, 16]. This relationship can vary by sex, type of activity, and other factors such as body image. In their study, Butt and colleagues [15] found that male adolescents had more fun than females in activities where they were able to exert the physical characteristics of PA. Body image accounted for significant sex differences, where females, but not males, reported body image improvements as a major reason for their PA participation and lack for time as a barrier to participation. The study also reported increasing sex differences in body image perceptions with age, where, as they got older, females became more self-aware of their body shape and appearance than males.

BWS refers to the discrepancy between one’s current/actual and ideal body image and body weight with scores of a lower magnitude suggesting satisfaction and scores of a higher magnitude suggesting dissatisfaction [17]. BWS is part of the broader concept of self-esteem but focuses on perceptions and attitudes about one’s bodyweight, physical appearance and to some extent, physical ability [18–20]. Research evidence has established relationships between body weight dissatisfaction, depression, negative self-evaluations, and low participation in PA [21,22,23]. However, little is known about how these factors affect PA behavior of youth living with human immunodeficiency virus (HIV+) compared to their HIV-negative (HIV-) counterparts. There is also limited information about whether PA behaviors of the two groups are similar. A few known studies of BWS among HIV+ individuals who are on antiretroviral therapy (ART) suggest that emotional challenges associated with peripheral fat loss and central fat gain are not uncommon [24,25]. Self-perceptions of body fat changes have also been associated with risks of lower adherence to ART [24]. Since regular participation in PA has been associated with improvements in physical self-perceptions across populations, it is conceivable this may have added benefits for individuals on ART, that include higher adherence to ART, greater sense of wellbeing, and increased PA levels.

Cultural perceptions of body image and body weight and their potential impact on enjoyment of PA and BWS cannot be ignored. Some studies suggest African societies are likely to associate thinness with ill-health and lower socio-economic status, while thinness might be viewed positively in most western societies [26,27]. This may vary by age group. A study of urban and rural adolescents in Botswana reported that overweight and obese adolescents were more dissatisfied with their weight and body proportions than those with optimal body weight [28]. Males had greater dissatisfaction with their muscle strength and muscle tone than females. Evidence from elsewhere in Africa suggests that adults prefer a bigger body size while the youth tend to prefer a leaner body size [29,30]. The evidence arouses some curiosity about the psychosocial determinants of PA and sedentary behavior among HIV+ youth on ART.

However, there is limited data on PA and sedentary behavior for children and youth living with HIV and on ART, specially in developing countries. This is despite the evidence that shows that individuals living with HIV are susceptible to debilitating psychological symptoms including chronic fatigue and anxiety [31], which can negatively affect PA behaviors. Since these and other factors have been associated with inactivity in the general population, they are equally likely to affect PA and sedentary behavior of individuals living with HIV. Investigations that examine if existing evidence on psychosocial determinants of PA and sedentary behavior of various youth populations equally applies to HIV+ youths would be highly informative. The evidence may expand existing knowledge and create a better understanding of the complex determinants of PA and sedentary behavior across populations.

To provide more context, the most recent global PA data among youth indicates that PA levels are generally below the evidence-based guidelines of 60 minutes of daily moderate-vigorous intensity activity [12,13]. However, a major limitation of this research, highlighted in a comprehensive review of the literature by Cortis et al. [8], is the focus on upper-middle to high income populations in Europe, North America, and Australia. These heavily studied populations tend to be similar on a variety of demographic variables, such as race, culture, income, health and wellbeing, and quality of life. By contrast, evidence from low to middle income countries, ethnic minorities, and individuals living with HIV remains limited. This lack of evidence impedes our ability to understand the extent to which relationships between PA and psychosocial variables are invariant relative to factors like socio-economic status and chronic illness.

In one large-scale study of 34 African countries, researchers examined PA and sedentary behaviors among adolescents aged 13–15 years old [32]. The researchers reported that across the 34 countries only 24% of the males and 15% of females met the World Health Organization (WHO) recommended PA levels. Amongst adolescents from Botswana, males spent an average of 1.5 active days per week compared to 1 day per week for females. Botswana was among seven countries where at least 25% of the males and 29% of the females spent 3 or more hours per day in sedentary behaviors like watching TV, talking with friends, and playing video games. The data confirm that regular PA in Botswana is lower than the recommended guidelines and suggests the importance of exploring variables that might be predictive of PA in this population. Furthermore, the descriptive data reported in this study ignores the potential effects of HIV status on PA behavior. Such information would be relevant to understanding overall health and wellbeing of youth in countries where high HIV prevalence rates may be a concern. Based on estimates from the Joint United Nations Programme on HIV/AIDS (UNAIDS) in 2018 [33], the HIV prevalence rate for Botswana youth age 15–24 years was 5.6% for males and 11.2% for females. About 84% of all infected youth were receiving antiretroviral therapy (ART). A previous PA and nutrition study involving children on ART indicated they participated less in PA (34). This evidence has significant implications for disease management and the overall health and wellbeing of this population. The prevalence rate also suggests a need for more investigations on PA and sedentary behavior as potential indicators of health-related quality of life in the affected population.

Limited data on the relationships among PASE, BWS, enjoyment of PA, and PA and sedentary behavior among youth in an African context makes it imperative to investigate these relationships. Given the existing evidence of a stigma that associates thinness with ill-health in Southern African populations, it will be interesting to examine variations in BWS and PA among HIV+ and HIV- youth. Such evidence is likely to offer a better understanding of how efficacy beliefs and BWS may affect or be affected by participation in PA among individuals living with HIV. The evidence may also be useful to the development of suitable strategies for the promotion of PA among people living HIV. Therefore, the purposes of this study were to:

- Determine if differences exist in daily PA, muscle strengthening activities, and sedentary behavior by HIV status and sex, controlling for age and BMI.
- Determine if psychosocial correlates of PA (PASE, BWS, and enjoyment of PA) differ as a function of HIV status, controlling for age, sex, and BMI.
- Examine if PASE, BWS, and enjoyment of PA are associated with daily PA, muscle strengthening activities, and sedentary behavior, controlling for age, sex, and BMI.

## Materials and methods

### Participants

A total of 250 HIV+ and HIV- youth (138 females) aged 12–23 years (Mean = 17.87, SD = 2.24) participated in the study. The HIV+ group (n = 88) was part of a previous 12-month ART and nutrition randomized control trial involving 201 children aged 5–12 years old [34,35]. Participants in this group were recruited into the first study at the point of their initial enrollment in an ART program offered by a pediatric clinic. That study ended 6 years prior to the current one. At the time of the current study, the group had made significant gains on overall health and wellbeing to the extent that they were comparable to their non-HIV infected counterparts on selected indicators. The HIV- group (n = 162; 94 female) was recruited from public junior and senior high schools in and around Gaborone the capital of Botswana. The decision to recruit the HIV- group from junior and senior high schools was to get youth of a comparable age-range to the HIV+ group from the previous study. Most of the HIV+ youth were at junior and senior high school.

### Procedures

After all the required ethical clearance had been obtained, the HIV+ group was contacted by phone and invited to participate in the study. The invitations were done through the pediatric clinic which had their contact information. A total of 108 out of the possible 201 participants responded to the phone calls and, of these, 88 agreed to take part in the current study. Twenty declined to participate because of constraints such as living in another town that was too far away from the study center. Ninety-two did not return the calls or the calls did not go through. The research team visited target schools to recruit participants to the HIV- group. Information about the study was passed through school counsellors. After participants and their parents gave consent and assent to take part in the study, as well as share the results from the HIV tests with the study, they were referred to the voluntary counselling and testing center for HIV screening. The HIV counselling and testing centers are manned by personnel who have been trained on HIV-related counselling and testing. Out of a total of 169 students recruited and referred for screening, seven declined to take part in the study, resulting in a 96% response rate. Study assessments were done on weekends at a youth facility in Gaborone. All participants completed questionnaires on their own, with the help of trained research assistants where necessary. The questionnaires were in English, which is the official language in Botswana.

### Measures

#### Demographics questionnaire

A demographic information questionnaire was used to gather such information as age, sex, and school level.

#### Anthropometric measurements

Anthropometric measures including height, weight, waist and hip circumferences were taken.

#### Daily physical activity and muscle strengthening

Daily PA and sedentary behavior were assessed using self-report items from the Youth Risk Behavior Surveillance System (YRBSS) [36]. Participants were asked to indicate how many days out of the past seven they were physically active for a total of 60 minutes in a day. The same was done for exercises aimed at strengthening or toning muscles, such as push-ups, sit-ups, and weightlifting. This variable was labelled muscle strengthening activities.

#### Sedentary behavior

Sedentary behavior was also assessed using self-report items from the YRBSS [36]. Participants were asked to indicate the number of hours in an average school day that they spent watching TV or playing videogames. The items were ranked from zero (I do not watch TV or play video games on an average school day) to seven (I watch TV or play video games 5 or more hours per day). The two items were combined and used as a measure of sedentary behavior. They were recoded from 1–3 with 1 representing no TV and Video watching, 2 representing .5-2hrs, and 3 representing more than 2 hrs of TV and video games per day. Daily PA, muscles strengthening behavior, and sedentary behavior items have been widely used in population-based surveys of children, adolescents, and young adults [13,37,38]

#### Body mass index (BMI)

Body weight was measured to the nearest 0.1 kg in light clothing using a portable Tanita digital scale (Tanita, Tokyo, Japan). Height was measured without shoes using a portable stadiometer (Seca, Hamburg, Germany) accurate to 1 mm. BMI was calculated (weight in kg/height in m^2^), and participants were assigned to the appropriate BMI category for age using the World Health Organization (WHO) classification: underweight (BMI < 18.50 kg/m^2^), normal weight (BMI 18.50–24.99 kg/m^2^), overweight (BMI ≥ 25 kg/m^2^), and obese (BMI ≥ 30 kg/m^2^) [39].

#### Body weight satisfaction

Body weight satisfaction (BWS) was determined as a congruence score between participants’ self-rating on body size (perceived body size) and their BMI classification based on WHO cut-off points [39]. This approach has been used successfully before in studies of body image [29, 40]. Perceived body size items ranged from 1 (underweight) to 4 (obese) and were adapted from the YRBSS [36]. BMI categories (underweight, normal weight, overweight, and obese) were represented by similar points (1 = underweight and 4 = obese). Congruence scores ranging between -3 and 3 were obtained by subtracting body size perception scores from BMI classifications. The absolute value of the BWS was also calculated so that perceptions of weighing less than actual and perceptions of weighing more than actual were considered to represent the same construct of having an inaccurate perception of weight.

#### Physical activity self-efficacy (PASE)

PASE was assessed using seven items adapted from a PA Self-Efficacy Scale [41]. The self-rating items ask participants to rate how confident they are, on a scale of 1–5, about their ability to be physically active during their free time even under various constraints. The constraints could be doing homework, watching TV, or playing video games. An example of the items is, “I know I can be physically active during my free time on most days” followed by five options ranging from “I cannot do this” (1) to “I am certain I can do this (5).” A test-retest reliability of a single factor solution of .84 from these items has been reported [41]. The data from the current study showed that a single factor based on the seven items had acceptable internal consistency with Cronbach’s α = .74. The usefulness of this unidimensional measure of self-efficacy has been previously supported [42]. Participants were also asked to rate their enjoyment of PA on a single item with a scale of 1 (very poor) to 5 (better than most).

### Ethical clearance

Permission to conduct this research was obtained from the Ethics Committees of the Ministry of Health in Botswana. IRB approvals were obtained from the collaborating institutions in Botswana. In addition to providing assent, all participants under 21 years had to provide signed consent from parents or legal guardians to participate in the study. Those aged 21 years and above, which is considered legal age of consent in Botswana, offered their own verbal and written consent. All the participants were required to provide verbal and signed assent/consent prior to completing questionnaires. Individuals and their parents/guardians were made aware of their rights to decline participation, withdraw from the study at any point, or decline to answer any questions.

### Statistical analyses

Descriptive statistics for demographic and various health indicators are presented, and independent samples t-tests and chi-square analyses were conducted to test for differences as a function of HIV status and sex. A multivariate analysis of variance (MANOVA) tested for simultaneous differences in a set of three dependent variables assessing physical activity behavior (daily PA, muscle strengthening activities, and sedentary behavior) by HIV status and sex. Given the potential age and BMI differences, these were included in the model as covariates. An examination of the Box’s M test showed that homogeneity of variance-covariance matrices was not violated. A second MANOVA was used to examine if PASE, BWS, and enjoyment of PA differ as a function of HIV status, controlling for age, sex, and BMI. A subsequent analysis was also conducted by repeating this MANOVA with the absolute value of BWS used as a dependent variable to identify if the direction of the discrepancy impacted the results. Multiple regression models were then run to test for the association of PASE, BWS, and enjoyment of PA with each of daily PA, muscle strengthening activities, and sedentary behavior. Age, sex, and BMI were entered simultaneously in the models to control for their effects on daily PA, muscle strengthening activities, and sedentary behavior. To avoid the inflated likelihood of a Type I error due to multiple testing, the significance level of each test (i.e., α) was adjusted using the Bonferroni correction that divides the significance level by the number of tests, resulting in .017 (= .05/3). An inspection of model assumptions indicated that linearity, normality, and multicollinearity assumptions were met for all model analyses. The data were analyzed using the Statistical Package for Social Sciences (SPSS) (Version 24).

## Results

Demographic and health variables were examined to consider differences as a function of HIV status and sex. The data showed that the groups were similar across many of these variables. However, the HIV- group was significantly younger than the HIV+ group. The HIV- group was also taller, and heavier than the HIV+ group, but there were no significant differences in BMI. The HIV+ had more cases of diabetes and asthma than the HIV- group. In this sample, the females were significantly younger than the males. They were also significantly shorter and lighter than the males but did not have significantly different BMI. A summary of this information is presented in Table 1.

**Table 1:** Demographic and health characteristics by HIV status and by sex .

Variable	HIV+ N = 88	HIV- N = 162	p-value	Males N = 111	Females N = 138	p-value	Total
Age (years), M ± SD	18 ± 1.74^a^	17.45 ± 2.36	^**b**^p < .001	18.31 ± 2.01	17.51 ± 2.34	^**b**^p = .005	17.87 ± 2.24
Height (cm), M ± SD	159.79 ± 9.18	162.71 ± 53.70	^**b**^p = .02	167.26 ± 10.53	157.21 ± 5.95	^**b**^p < .001	161.69 ± 9.69
Weight (kg), M ± SD	49.26 ± 6.97	53.70 ± 10.29	^**b**^p < .001	54.5 ± 8.51	50.25 ± 9.85	^**b**^p < .001	52.15 ± 9.49
BMI (kg/m^2^), M ± SD)	19.37 ± 3.08	20.33 ± 4.07	^**b**^p = .06	19.62 ± 3.85	20.30 ± 3.70	^**b**^p = .156	19.99 ± 3.78
BMI Categories*			^c^p = .276			^c^p = .123	
1 Underweight	40 (45%)	65 (40%)		54 (48%)	51 (37%)		105 (42%)
2 Normal weight	45 (51%)	84 (52%)		53 (48%)	76 (55%)		129 (52%)
3 Overweight	1 (1%)	10 (6%)		2 (2%)	9 (7%)		11 (4%)
4 Obese	1 (1%)	3 (2%)		2 (2%)	2 (1%)		4 (2%)
Diabetes, n (%)	18 (21%)	11 (7%)	^c^p = .01	12 (11%)	17 (13%)	^c^p = .76	29 (12%)
Hypertension, n (%)	0	1 (.6%)	^c^p = .45	1 (.9%)	0	^c^p = .26	1 (.4%)
Asthma, n (%)	10 (11%)	6 (4%)	^c^p = .03	9 (8%)	7 (5%)	^c^p = .31	16 (6%)
Chest pains, n (%)	4 (5%)	2 (1%)	^c^p = .12	4 (4%)	2 (1%)	^c^p = .26	6 (2%)
Bone injury, n (%)	8 (9%)	16 (10%)	^c^p = .75	6 (6%)	18 (13%)	^c^p = .05	24 (10%)
Allergies, n (%)	9 (10%)	9 (6%)	^c^p = .21	9 (8%)	9 (7%)	^c^p = .59	18 (7%)
Advised not to do physical activity, n (%)	1 (1%)	1 (.6%)	^c^p = .68	1 (.9%)	1 (.7%)	^c^p = .86	2 (.8%)

Note

^a^ Mean ± SD

^b^ Independent samples t-test

^c^ Pearson Chi-Square test

*percentages do not add up to 100% because of missing data.

### Daily PA, muscle strengthening activities, and sedentary behavior by HIV status and sex

To address the first objective of the study, a two-way MANOVA was run to determine differences in daily PA, muscle strengthening activities, and sedentary behavior by HIV status and sex, controlling for age and BMI. The results indicated that there was not a significant interaction effect, *F*(3,232) = 1.36, *p*>.05. However, there were significant main effects for HIV status, *F*(3,232) = 3.18, *p* < .05, $\eta_{p}^{2}$ = 0.04, and sex, *F*(3,232) = 12.27, *p* < .05, $\eta_{p}^{2}$ = 0.14, which will be discussed because of limited research in this area and the relevance of these variables to the study’s objectives. An examination of univariate results indicted that the significant differences for HIV status were evident for daily PA, *p* < .05, and for muscle strengthening activities, *p* < .01. The mean differences showed that the HIV- group (*M* = 1.20, *SE* = 0.06, *CI* = 1.08 to 1.32) had significantly higher daily PA than the HIV+ group (*M* = 0.99, *SE* = 0.08, *CI* = 0.82 to 1.15*)*. The HIV- group (*M* = 0.91, *SE* = 0.06, *CI* = 0.79 to 1.03) also had a significantly higher mean for muscle strengthening activities than the HIV+ group (*M* = 0.63, *SD* = 0.08, *CI* = 0.47 to 0.78*)*. Univariate analyses for sex indicated that the only significant effect was for muscle strengthening activities with males (*M* = 1.05, *SE* = 0.07, *CI* = 0.91 to 1.20) reporting more muscle strengthening activities than females (*M* = 0.48, *SE =* 0.07, *CI* = 0.34 to 0.61). A summary of the mean differences by HIV status and sex is presented in Table 2.

**Table 2:** Means and standard errors for daily PA, muscle strengthening and sedentary behavior by HIV status and sex.

Variable	HIV+ N = 87	HIV- N = 153	*p*-value^a^	Males N = 106	Females N = 134	*p*-value^b^	HIV+ Males N = 43	HIV+ Females N = 44	HIV- Males N = 63	HIV- Females N = 90	*p*-value^c^
Daily PA	0.99 ± 0.082	1.20 ± 0.062	*p* = .045	1.19 ± 0.075	1.00 ± 0.069	*p* = .064	1.15 ± 0.12	0.82 ± 0.11	1.22 ± 0.094	1.17 ± 0.0.81	*p* = .160
MSA*	0.63 ± 0.080	0.91 ± 0.060	*p* = .006	1.05 ± 0.073	0.48 ± 0.067	*p* < .001	0.94 ± 0.11	0.31 ± 0.11	1.16 ± 0.092	0.65 ± 0.080	*p* = .529
Sedentary	1.02 ± 0.071	0.92 ± 0.053	*p* = .246	1.04 ± 0.90	0.90 ± 0.059	*p* = .135	1.14 ± 0.10	0.90 ± 0.10	0.93 ± 0.081	0.91 ± 0.070	*p* = .211

Note

^a^ ANOVA main effect for HIV status

^b^ ANOVA main effect for sex

^c^ ANOVA sex x HIV status Interaction

*Muscle Strengthening Activities

### Psychosocial factors, Daily PA, muscle strengthening, sedentary behavior and HIV Status

The MANOVA indicated that there was a statistically significant multivariate main effect for HIV status, *F*(3, 233) = 6.49, *p*< .001, $\eta_{p}^{2}$ = .08. The univariate results indicated that this effect was significant for PASE, *p* < .001, and for BWS, *p* < .05. An examination of means for PASE by HIV status showed a higher mean for HIV- participants (*M* = 3.58, *SE* = 0.07, *CI* = 3.44 to 3.72) compared to HIV+ participants (*M* = 3.11, *SE* = 0.09, *CI* = 2.93 to 3.30). Results for BWS indicated that HIV- participants (*M* = -0.24, *SE* = 0.05, *CI* = -0.35 to -0.14) reported a more negative BWS than did HIV+ participants (*M* = -0.03, *SE* = 0.07, *CI* = -0.18 to 0.11).

A subsequent MANOVA was conducted using the absolute value of BWS instead of BWS to see if the previous finding was driven by the direction of the discrepancy rather than the magnitude of the discrepancy. The results yielded a statistically significant multivariate main effect for HIV status, *F*(3, 233) = 5.80, *p* < .01, $\eta_{p}^{2}$ = .07. However, the univariate results indicated that this effect was only significant for PASE, *p* < .001.

Multiple regression analyses were run to address the third of objective of this study regarding whether PASE, BWS, and enjoyment of PA are associated with daily PA, muscle strengthening activities, and sedentary behavior, controlling for age, sex, and BMI. Age, sex, and BMI were simultaneously entered in model 1 of the regression followed by the three psychosocial correlates in model 2. The results are summarized in Table 3.

**Table 3:** Summary of multiple regression results.

Variable	*B*	*SE*_*B*_	*β*
**Daily PA**			
PASE	0.25	0.06	0.28***
BWS	0.03	0.08	0.03
Enjoyment of PA	0.08	0.04	0.14*
Sex	-0.07	0.10	-0.05
Age	-0.02	0.02	-0.06
BMI	0.01	0.01	0.07
**Muscle strengthening activities**			
PASE	0.23	0.06	0.25***
BWS	-0.07	0.08	-0.05
Enjoyment of PA	0.09	0.04	0.16*
Sex	-0.48	0.09	-0.30***
Age	-0.05	0.02	-0.14*
BMI	0.00	0.01	-0.00
**Sedentary behavior**			
PASE	0.04	0.05	0.05
BWS	0.10	0.07	0.09
Enjoyment of PA	0.05	0.03	0.11
Sex	-0.06	0.09	-0.04
Age	0.06	0.02	0.20**
BMI	0.00	0.01	0.00

Note.

***p < .001

**p< .01

*p< .05; **B** = Unstandardized Coefficient

***SE***_***B***_ = Standard error; ***β =*** Standardized Coefficient

After controlling for age, sex, and BMI in model 1, PASE, BWS, and enjoyment of PA explained a significant portion of the variance in daily PA, *F*(6, 233) = 6.55, *p* < .001, adjusted *R*^2^ *=* .13. Higher PASE (*B* = .25, 95% CI [.13, .36], *β* = .29, *p<* .001) and greater enjoyment of PA (*B* = .08, 95% CI [.00, .15], *β* = .14, *p<* .01) were predictive of higher daily PA. Both model 1 (age, sex, and BMI), *F*(3, 236) = 11.15, *p* < .001, adjusted *R*^2^ = .11, and model 2 (PASE, muscle strengthening activities, BWS), *F*(6, 233) = 12.60, *p* < .001, adjusted *R*^2^ = .23, were statistically significant predictors of muscle strengthening activities. Males reported more muscle strengthening activities than females (*B* = -.48, 95% CI [-.67, -.30], *β =* -.29, *p <* .001), while an increase in age was associated with a decline in muscle strengthening activities (*B* = -.05, 95% CI [-.09, -.01], *β =* -.14, *p =* .01). Higher PASE (*B* = .23, 95% CI [.12, .34], *β =* .25, *p <* .001) and enjoyment of PA (*B* = .09, 95% CI [.02, .15], *β =* .16, *p <* .02) were predictive of greater participation in muscle strengthening activities. Only the covariates were significant predictors of variance in sedentary behaviors, *F*(3, 236) = 4.12, *p* < .01, adjusted *R*^2^ = .05. Age significantly predicted sedentary behavior (*B* = .06, 95% CI [.02, .10], *β* = .20, *p <* .01), indicating that older age was associated with more sedentary behavior.

## Discussion

There is limited research evidence related to psychosocial correlates and determinants of PA and sedentary behavior among youth who are not middle or upper-class and from western countries. Furthermore, there is no evidence on these relationships among adolescents living with HIV. Given the known benefits of PA participation to overall health and wellbeing, such information would be valuable to PA and health promotion among this group. Therefore, we sought to examine if daily PA, muscle strengthening activities, and sedentary behavior differ by HIV status and sex while controlling for age and BMI. Second, we wanted to determine if psychosocial correlates of PA (PASE, BWS, and enjoyment of PA) differ as a function of HIV status while controlling for age, sex and BMI. Finally, we wanted to determine if psychosocial factors are associated with participation in daily PA, muscle strengthening activities, and sedentary behavior among youth in our study while controlling for their age, sex, and BMI.

Descriptive data shows that except for being shorter, lighter, older, and reporting more cases of diabetes and asthma, the HIV+ group did not differ significantly from the HIV- group on selected demographic variables and indicators of overall health and wellbeing. Importantly, given the differences in height, weight, and age, all analyses were conducted controlling for BMI and age.

Results from this study show that males spent significantly more time in muscle strengthening activities than females. They also reported a marginally higher involvement in daily PA compared to females. These significant sex differences are noteworthy because they are consistent with previous findings on PA and muscle strengthening activities [12,13,32]. Previous studies observed that PA disparities between males and females are due to a myriad of factors. Many of these are related to policy limitations when it comes to the promotion of PA among females, safety concerns for females, and limited access and opportunities for females. There is also gender role socialization and persistent sociocultural perceptions of PA as a male domain, which is more prevalent in some countries compared to others [43]. Anecdotal evidence suggests Botswana is among countries where cultural perceptions of gender roles and sport socialization reinforce sex disparities in sport and PA participation. For instance, participants in structured and less structured neighborhood soccer programs are overwhelmingly male, while similar program opportunities are extremely limited for females. Higher participation in muscle strengthening activities are among males compared to females is also noteworthy. The differences could be explained in terms of sex-based preferences for muscle strengthening activities, as well as, cultural or contextual reinforcement of these behaviors and attitudes. It could be that a social narrative that associates muscularity and physical strength with a greater sense of maleness encourages more males to engage in weightlifting and muscle development activities compared to females [44]. This may also be an outcome as well as predictor of perceived efficacy (PASE) regarding muscle strengthening activities. The findings suggest addressing structural and socio-cultural constraints to participation in PA and muscle strengthening activities remains key to reducing sex disparities.

No significant differences were found on sedentary behavior by sex or HIV status, however, sedentary behavior significantly increased with age. This finding is consistent with what has been reported about the growing prevalence of sedentary behavior across children and youth populations [12,43]. It also means this problem cuts across sex and HIV status. This is not surprising given that the HIV+ and HIV- groups in this study had similar profiles as demonstrated by selected demographic variables and indicators of overall health and well-being. Findings on sex differences in sedentary behavior, however, have been inconsistent. Guthold et al. [32] found no sex differences in sedentary behavior in 31 of the 34 countries they studied, but Botswana was one of the few where females were found to be more sedentary than males. This finding was not supported by our data. It could be that our study had a smaller and narrower sample of youth compared to the sample described by Guthold and colleagues.

Results from this study showed that the HIV- group had higher daily PA, muscle strengthening activities and PASE, but lower BWS than the HIV+ group. The results also showed a positive relationship between PASE, enjoyment of PA and higher participation in daily PA and muscle strengthening activities. This suggests improvements in PASE might benefit PA behavior, especially among HIV+ youth. The positive relationship between PASE and higher participation in daily PA and muscle strengthening activities confirms findings from previous studies, which suggests that attitudes and efficacy beliefs are indeed important predictors as well as mediators of PA behavior [9,26,42,45]. This study supports the facilitative role of higher self-efficacy in PA participation reported in a systematic review of literature by Cortis et al. [8]. Cortis and colleagues reported that a convincing positive association between self-efficacy and PA was evident in 45% of the reviews that explored this relationship. Similarly, Dzewaltowski et al. found that even greater perception of PA opportunities was associated with higher PA self-efficacy [42]. Higher PASE and higher engagement in daily PA and muscle strengthening activities among the HIV- group compared to the HIV+ group is noteworthy. Given what is known about the centrality of experience and task mastery as dependable sources of efficacy beliefs [4,5], it is possible that lower PA levels in the HIV+ group would have led to lower perceived efficacy about the group’s capacity to engage in PA and vice versa. Low PA and fitness in HIV+ in children on ART have been previously reported [46]. Understanding why this happens deserves more attention because it has implications on PA decline in adulthood.

Although, this study’s findings on the direction and magnitude of BWS were not consistent, they may have implications relative to body image and body dissatisfaction issues reported in previous studies [26,44,47,48]. First, the current findings support conclusions from the body image literature regarding the complexity of determining BWS in adolescents and youth populations [43,45]. The observed difference between the groups suggest a possible context specific association of thinness with ill-health reported previously (40). Considering the overall low prevalence of overweight and obesity in this sample of youth, and the fact that the HIV- group had a higher BMI score (although not statistically significant) and had fewer underweight cases compared to the HIV+ group, it is plausible that the higher BWS in the HIV- group simply represented a preference for a larger or heavier body that wasn’t there for the HIV+ group. The body weight discrepancy might have been of lesser concern to HIV+ participants because they have gotten used to having smaller and lighter bodies as a consequence of their long-term health condition. Earlier findings from this group showed that they had stunting and were generally underweight (35). Caution is advised regarding this directional interpretation of BWS because the discrepancy between the actual and ideal weight has been found to go in either direction as a result of various factors not examined in this study, such as, self-esteem, family factors, and mental health [45]. This would make preference for thinness among adolescents and youth reported elsewhere [45] a less likely explanation for the body weight dissatisfaction observed in this study.

The current findings suggest that more research is needed to understand the role of psychosocial factors like PASE, BWS, and enjoyment of PA on PA and health behaviors of youth living with HIV. The differences on daily PA and muscle strengthening activities between HIV+ and HIV- participants suggest more research is needed to understand the extent to which the disease and being on ART limits or alters the trajectories of PA and muscle strengthening activities for individuals living with HIV. Another important consideration is the impact of culture, gender roles, and body stereotypes on PA behaviors for individuals living with HIV and on ART. Evidence of emotional challenges associated with peripheral fat loss and central fat gain due to ART, reported elsewhere, suggest this is an issue worthy of investigation [24]. An exploration of possible intersections with cultural perceptions of body image and BWS will also be interesting [29]. Investigating these complex relationships requires more robust designs, such as randomized control trials, mixed methods, and longitudinal designs.

There are several limitations worth noting in the current study. First the cross-sectional nature of our study means we cannot determine a causal relationship between study variables. Although we have discussed these findings as suggesting that PASE and enjoyment of PA predict PA and muscle strengthening activities, the opposite is equally possible. Although the HIV+ group was assessed in a previous study in which they were followed for a few years, data from this study is truly cross-sectional. A time series longitudinal design allowing periodic assessments of this group and their counterparts who are HIV- would certainly allow for stronger conclusions. This line of research could also benefit from experimental and mixed-method designs.

Another limitation of our study is that our data is based solely on self-report measures. Given the known limitations of self-reported PA, the use of objective measures of PA and muscle strengthening activities is likely to enhance the quality of PA assessment. Although the YRBSS and the PASE items have been used quite extensively and produced highly consistent results with different sub-populations, there may be cultural and other contextual limitations to these tools that may affect the quality of responses. Furthermore, we cannot rule out bias from recall and social desirability. Finally, investigations that examine other possible barriers and facilitators of PA, sedentary behavior, and enjoyment of PA in this context and among HIV+ youth are likely to provide additional insights. Objective measures may be needed to verify self-report specially to determine muscular strengthening, enjoyment of PA and BWS. The role of social influencers such as parents, peers, school, and the community should offer a more comprehensive picture of the PA environment. The weak result on enjoyment of PA in the study, when it is generally associated with increased PA and muscle strengthening activities, suggests a need for a more robust measure of this variable.

## Conclusions

Overall the current study demonstrates the relationship between PASE, daily PA and muscle strengthening activities. Further, the study shows the potential role that PASE, enjoyment of PA, and BWS could play in the promotion of PA among HIV+ youth. The findings have implications for interventions and clinical research into how PA and exercise could be used to mitigate the effects of living with chronic conditions such as HIV. The findings suggest the possibility of some socio-cultural nuances related to definitions of body weight, body size, and enjoyment of PA that are worthy of further investigation. Studying such nuances may be necessary to understand and develop appropriate strategies to promote healthy PA behaviors and reduce barriers to PA and exercise. One of the strengths of this study is that it offers much needed evidence on correlates of PA and sedentary behavior among a less studied subpopulation of youth. These are HIV+ youth on ART and from an African country. It also makes a comparison of these youth with those who are HIV-. The comparative evidence provided in this study is particularly relevant to the development of a global understanding of PA as it relates to health and wellbeing of youth across the board, especially those living with HIV and other chronic conditions.

## Supporting information

S1 Dataset HIV PA Efficacy and BWS. (SAV)

## References

[1] Haugen T, Säfvenbom R, Ommundsen Y. Physical activity and global self-worth: The role of physical self-esteem indices and sex. Mental Health and Physical Activity, 2011, 4(2): 49–56. 10.1016/j.mhpa.2011.07.001.

[2] Bauman AE, Reis RS, Sallis JF, Wells JC, Loos RJ., Martin BW. Correlates of physical activity: why are some people physically active and others not? The Lancet, 2012, 380(9838): 258–71. 10.1016/S0140-6736(12)60735-1 22818938

[3] Sollerhed AC, Apitzsch E, Råstam L, Ejlertsson G. Factors associated with young children's self-perceived physical competence and self-reported physical activity. Health Education Research, 2007, 23(1): 125–136. 10.1093/her/cym010 17347524

[4] Bandura A. Self-efficacy: The exercise of control. New York: W.H., 1997.

[5] Bandura A. Social cognitive theory: an agentic perspective. Annual Review of Psychology, 2001, 52, 1–26. 10.1146/annurev.psych.52.1.1 11148297

[6] World Health Organization. Global Action Plan on Physical Activity 2018–2030: More Active People for Healthier World,2018, Geneva, Switzerland: Author. Retrieved from http://apps.who.int/iris/bitstream/handle/10665/272722/9789241514187-eng.pdf.

[7] Bauman AE, Reis RS, Sallis JF, Wells JC, Loos RJ., Martin BW. Correlates of physical activity: why are some people physically active and others not? The Lancet, 2012, 380(9838): 258–71. 10.1016/S0140-6736(12)60735-1 22818938

[8] Cortis C, Puggina A, Pesce C, et al Psychological determinants of physical activity across the life course: A" DEterminants of DIet and Physical ACtivity"(DEDIPAC) umbrella systematic literature review. PloS one, 2017, 12(8): e0182709 10.1371/journal.pone.0182709 28817676 PMC5560721

[9] Manley D, Cowan P, Graff CG, Perlow M, Rice P, Richey P, Zoila Sanchez Z. Self-efficacy, physical activity, and aerobic fitness in middle school children: Examination of a pedometer intervention program. Journal of Pediatric Nursing, 2014, 29(3): 228–237. 10.1016/j.pedn.2013.10.011 24263251

[10] Phillips EA, Comeau DL, Pisa PT, Stein AD, Norris SA. Perceptions of diet, physical activity, and obesity-related health among black daughter-mother pairs in Soweto, South Africa: a qualitative study. BMC Public Health, 2016, 16(1): 750 10.1186/s12889-016-3436-8 27506678 PMC4977727

[11] Verloigne M, Ridgers ND, Chinapaw M, et al Patterns of objectively measured sedentary time in 10-to 12-year-old Belgian children: an observational study within the ENERGY-project. BMC pediatrics, 2017, 17(1): 147 10.1186/s12887-017-0894-9 28615079 PMC5471712

[12] Tremblay MS, Carson V, Chaput JP, et al Canadian 24-hour movement guidelines for children and youth: an integration of physical activity, sedentary behaviour, and sleep. Applied Physiology, Nutrition, and Metabolism, 2016, 41(6): S31a1–S327. 10.1139/apnm-2016-0151 27306430

[13] World Health Organization. Global Recommendations on Physical Activity for Health. 2010, Geneva, Switzerland: Author. Retrieved from http://apps.who.int/iris/bitstream/handle/10665/44399/9789241599979_eng.pdf;jsessionid=48F0A977DBFEDAC153B13DEE89A9A103?sequence=1 26180873

[14] Michael SL, Coffield E, Lee SM, Fulton JE. Variety, enjoyment, and physical activity participation among high school students. Journal of physical activity and health, 2016, 13(2): 223–230. 10.1123/jpah.2014-0551 26107142 PMC5295133

[15] Butt J, Weinberg RS, Brecken JD, Claytor RP. Adolescent physical activity participation and motivational determinants across sex, age, and race. Journal of Physical Activity and Health, 2011, 8(8), 1074–1083. https://doi-org.proxy1.cl.msu.edu/10.1123/jpah.8.8.1074 22039125 10.1123/jpah.8.8.1074

[16] Motl RW, Dishman RK, Saunders RP, et al Measuring enjoyment of physical activity in adolescent girls. American Journal of Preventive Medicine, 2001, 21(2):110–117. 10.1016/s0749-3797(01)00326-9 11457630

[17] Cash T F, Pruzinsky T. Body Image: A handbook of theory, research, and clinical practice The Guilford Press, 2004.

[18] Cash T F, Szymanski M L. The development and validation of the Body-Image Ideals Questionnaire. Journal of personality assessment, 1995, 64(3): 466–477. 10.1207/s15327752jpa6403_6 16367722

[19] Cash TF, Smolak L. Body Image: A handbook of science, practice, and prevention Guilford Press, 2011.

[20] Grogan S. Body image: Understanding body dissatisfaction in men, women and children Routledge/Taylor & Francis Group, 2008.

[21] Hausenblas HA, Campbell A, Menzel JE, et al Media effects of experimental presentation of the ideal physique on eating disorder symptoms: A meta-analysis of laboratory studies. Clinical Psychology Review, 2013, 33(1): 168–181. 10.1016/j.cpr.2012.10.011 23232051

[22] Dion J, Blackburn ME, Auclair J, et al Development and aetiology of body dissatisfaction in adolescent boys and girls. International journal of adolescence and youth, 2015, 20(2): 151–166. 10.1080/02673843.2014.985320 25931646 PMC4391290

[23] Jones LR, Fries E, Danish SJ. Gender and ethnic differences in body image and opposite gender figure preferences of rural adolescents. Body image, 2007, 4(1): 103–108. 10.1016/j.bodyim.2006.11.005 18089257 PMC2031852

[24] Plankey M, Bacchetti P, Jin C, et al Self-perception of body fat changes and HAART adherence in the Women’s Interagency HIV Study. AIDS and Behavior, 2009, 13(1): 53 10.1007/s10461-008-9444-7 18688706 PMC2902995

[25] Wilkins ML, Dallas RH, Porter JS, et al Characterizing body image in youth living with HIV. AIDS and Behavior, 2016, 20(8): 1585–1590. 10.1007/s10461-015-1271-z 26721247

[26] Sonneville KR, Calzo JP, Horton NJ, et al Body satisfaction, weight gain and binge eating among overweight adolescent girls. International Journal of Obesity, 2012, 36(7): 944 10.1038/ijo.2012.68 22565419 PMC3394875

[27] Swami V, Frederick DA, Aavik T, et al The attractive female body weight and female body dissatisfaction in 26 countries across 10 world regions: Results of the International Body Project I. Personality and social psychology bulletin, 2010, 36(3): 309–325. 10.1177/0146167209359702 20179313

[28] Malete L, Motlhoiwa, Shaibu BK, et al Body Image Dissatisfaction is increased in male and overweight/obese adolescents in Botswana. Journal of Obesity, 2013, 10.1155/2013/763624. PMC3619546 23634296

[29] Naigaga DA, Jahanlu D, Claudius HM, et al Body size perceptions and preferences favor overweight in adult Saharawi refugees. Nutrition journal, 2018, 17(1): 17 10.1186/s12937-018-0330-5 29426331 PMC5807821

[30] Rguibi M, Belahsen R. Body size preferences and sociocultural influences on attitudes towards obesity among Moroccan Sahraoui women. Body Image, 2006, 3(4): 395–400. 10.1016/j.bodyim.2006.07.007 18089243

[31] Barroso J, Hammill BG, Leserman J, et al Physiological and psychosocial factors that predict HIV-related fatigue. AIDS and Behavior, 2010, 14(6): 1415–1427. 10.1007/s10461-010-9691-2 20352317 PMC2975810

[32] Guthold R, Cowan MJ, Autenrieth CS, Kann L, Riley LM. Physical activity and sedentary behavior among schoolchildren: a 34-country comparison. The Journal of pediatrics, 2010, 157(1): 43–49. 10.1016/j.jpeds.2010.01.019 20304415

[33] UNAIDS. HIV prevalence among young people (15–24). 2018, October 15, Retrieved from http://aidsinfo.unaids.org/.

[34] Malete L, Mokgatlhe L, Nnyepi M, et al Effects of a High Protein Food Supplement on Physical Activity, Motor Performance and Health Related Quality of Life of HIV Infected Botswana Children on Anti-Retroviral Therapy (ART). AIMS Public Health, 2017, 4 (3): 258–277. 10.3934/publichealth.2017.3.258 29546216 PMC5690453

[35] Nnyepi M, Bennink MR, Jackson-Malete J, et al Nutrition status of HIV+ children in Botswana. Health Education, 2015, 115(5): 495–514. 10.1108/HE-04-2014-0052

[36] Kann L, Kinchen S, Shanklin SL, et al Youth risk behavior surveillance system—United States, 2013. Morbidity and Mortality Weekly Report: Surveillance Summaries, 2014, 63(4): 1–168. 22673000

[37] Lowry R, Michael S, Demissie Z, Kann L, Galuska DA. Associations of physical activity and sedentary behaviors with dietary behaviors among US high school students. Journal of obesity, 2015, 1–8. 10.1155/2015/876524. PMC4458549 26101666

[38] Centers for Disease Control and Prevention. Youth risk behavior surveillance: United States, 2011. Morbidity and Mortality Weekly Report, 2012, 61(4):1–162. 22673000

[39] de Onis M, Lobstein T. Defining obesity risk status in the general childhood population: which cut‐offs should we use? International Journal of Pediatric Obesity, 2010, 5(6): 458–460. 10.3109/17477161003615583 20233144

[40] Brown C, Shaibu S, Maruapula S, Malete L, Compher C. Perceptions and attitudes towards food choice in adolescents in Gaborone, Botswana. Appetite, 2015, 95: 29–35. 10.1016/j.appet.2015.06.018 26148457

[41] Dishman RK, Motl RW, Saunders R, et al Enjoyment mediates effects of a school-based physical-activity intervention. Medicine and science in sports and exercise, 2005, 37(3): 478–87. 10.1249/01.mss.0000155391.62733.a7 15741848

[42] Dzewaltowski DA, Geller KS, Rosenkranz RR, Karteroliotis K. Children's self-efficacy and proxy efficacy for after-school physical activity. Psychology of Sport and Exercise, 2010, 11(2): 100–106. 10.1016/j.psychsport.2009.08.001.

[43] Arundell L, Fletcher E, Salmon J, Veitch J, Trina Hinkley T. The correlates of after-school sedentary behavior among children aged 5–18 years: a systematic review. BMC Public Health, 2015, 16(1): 58 10.1186/s12889-015-2659-4 26795731 PMC4722784

[44] Lubans DR, Cliff DP. Muscular fitness, body composition and physical self-perception in adolescents. Journal of Science and Medicine in Sport, 2011, 14(3): 216–221. 10.1016/j.jsams.2010.10.003 21111677

[45] Kołoło H, Guszkowska M, Mazur J, Dzielska A. Self-efficacy, self-esteem and body image as psychological determinants of 15-year-old adolescents’ physical activity levels. Human Movement, 2012, 13(3): 264–270. 10.2478/v10038-012-0031-4

[46] Somarriba G, Lopez-Mitnik G, Ludwig DA, et al Physical fitness in children infected with the human immunodeficiency virus: associations with highly active antiretroviral therapy. AIDS research and human retroviruses, 2013, 29(1): 112–120. 10.1089/AID.2012.0047 22747252 PMC3537323

[47] McCabe MP, Ricciardelli LA. Body image dissatisfaction among males across the lifespan: A review of past literature. Journal of Psychosomatic Research, 2004, 56: 675–685. 10.1016/S0022-3999(03)00129-6 15193964

[48] Solomon-Krakus S, Sabiston C, Brunet J, Andree L. et al Body image self-discrepancy and depressive symptoms among early adolescents. Journal of Adolescent Health, 2017, 60: 38–43 10.1016/j.jadohealth.2016.08.024 27793726

